# Left Main Snorkel Stent Thrombosis in Association With Takayasu Arteritis

**DOI:** 10.7759/cureus.63761

**Published:** 2024-07-03

**Authors:** Aleksan Khachatryan, Vahagn Tamazyan, Margarita Sargsyan, Reyaz U Haque, Tufan Cinar, Joel Alejandro, Hakob Harutyunyan, Ashot Batikyan

**Affiliations:** 1 Department of Internal Medicine, University of Maryland Medical Center, Midtown Campus, Baltimore, USA; 2 Department of Internal Medicine, Maimonides Medical Center, New York, USA; 3 Department of Cardiology, "Heratsi" Hospital Complex № 1, Yerevan, ARM; 4 Department of Cardiovascular Medicine, University of Maryland Medical Center, Midtown Campus, Baltimore, USA; 5 Department of Internal Medicine, North Central Bronx Hospital, New York, USA

**Keywords:** mra, coronary artery bypass grafting(cabg), restenosis, ostial stenosis, snorkel stent, cardiac arrest, stent thrombosis, primary percutaneous coronary intervention (pci), left main stenosis, takayasu arteritits

## Abstract

Takayasu arteritis (TA) is a rare form of large vessel arteritis that predominantly affects the aorta and its major branches. This inflammation leads to thickening, fibrosis, and stenosis of the arterial walls, which may lead to thrombus formation. The resulting symptoms are typically due to ischemia of the end organs. Coronary artery involvement is uncommon and primarily affects the ostia of the arteries. Ostial involvement of the coronary arteries can have a dramatic course, including fatal outcomes.

We present the case of a 16-year-old female with TA involving the ostium of the left main coronary artery, causing severe stenosis. A successful percutaneous coronary intervention was performed on the left main artery with snorkel stent placement, which was complicated by cardiac arrest seven months later due to complete thrombosis of the proximal opening of the protruding stent.

## Introduction

Takayasu arteritis (TA) is a chronic, granulomatous inflammatory disease primarily affecting large arteries such as the aorta and its major branches. This condition is characterized by thickening, fibrosis, and stenosis of the arterial walls, with or without subsequent thrombus formation, leading to ischemic events. Consequently, TA has also been termed "pulseless disease," "occlusive thrombo-aortopathy," or "aortic arch syndrome." More severe inflammation can affect the arterial media, potentially causing aneurysms. The exact etiology and pathophysiology of the disease remain unknown. Manifestations include nonspecific constitutional complaints as well as symptoms related to end-organ ischemia.

Cardiac presentations of TA include pericarditis, myocarditis, valvular insufficiency, dilated cardiomyopathy, and myocardial infarction [1]. Coronary artery involvement occurs in 9-10% of cases and significantly contributes to mortality and morbidity [2,3]. It is postulated that the mechanism of coronary artery involvement is the extension of the inflammatory process from the aorta into the opening and proximal segments of the coronaries, causing intimal hyperplasia, contracture, and stenosis. The most common coronary lesion in TA is ostial stenosis [4]. Given the ostial nature of the coronary lesions, management presents a significant challenge.

Accordingly, the case described below highlights the challenges in managing ostial left main (LM) artery stenosis and associated complications in the context of TA, emphasizing the potential advantages of surgical revascularization.

## Case presentation

A 16-year-old female with a medical history of iron deficiency anemia and severe acne vulgaris presented with a burning sensation in her chest.

The symptom began approximately seven months prior, following the initiation of erythromycin for a long-standing wound on her left hip. Initially, the burning sensation was attributed to pill esophagitis. However, the condition progressively worsened, and the patient began experiencing dyspnea on exertion. She denied any paroxysmal nocturnal dyspnea, orthopnea, leg swelling, syncope, presyncope, headache, lightheadedness, claudication, nausea, vomiting, joint pain, constipation, diarrhea, oral ulcers, fevers, muscle aches, or photosensitivity.

The patient's medical history dates back two years when she developed severe chest and back acne, for which she was treated with high doses of isotretinoin and doxycycline. Following the treatment of her acne, she developed a small rash on the left hip area, which progressed into an open wound with periods of flares and remissions. Initially, she was treated with doxycycline and later switched to erythromycin.

Apart from the medications used for acne and rash treatment, the patient has not been on any other medications. She denied any family history of autoimmune conditions. Her mother had a history of mitral valve prolapse.

Upon presentation, the vital signs were as follows: temperature 37°C, blood pressure 124/78 mmHg, heart rate 97 beats per minute, respiratory rate 16 breaths per minute, and oxygen saturation 99% on room air. The physical examination, including the cardiovascular assessment, was unremarkable except for an erythematous indurated plaque on the left hip.

The pertinent laboratory results related to this presentation are summarized in Table 1.

**Table 1:** Results of laboratory workup WBC: white blood cell; CRP: C-reactive protein; ESR: erythrocyte sedimentation rate; LDL: low density lipoprotein; ANA: antinuclear antibody; ANCA: antineutrophil cytoplasmic antibody; UA: urine analysis; HIV: human immunodeficiency virus; SARS-CoV-2: severe acute respiratory syndrome coronavirus 2

Parameter	Results	Reference range
WBC	8.1 K/mcL	4.5 - 13.0 K/mcL
Hemoglobin	10.3 g/dL	12.0 - 16.0 g/dL
CRP	1.6 mg/dL	≤1.0 mg/dL
ESR	23 mm/hour	<20 mm/hour
LDL	65 mg/dL	<100 mg/dL
ANA IgG	None detected	None detected
Serine Protease 3 IgG	3 AU/mL	0 - 19 AU/mL
IgG	1341 mg/dL	810 - 1,792 mg/dL
IgG4	42 mg/dL	2 - 170 mg/dL
Myeloperoxidase (MPO) Ab	1 AU/mL	0 - 19 AU/mL
ANCA	<1:20	<1:20
C3 Complement	121 mg/dL	88 - 165 mg/dL
C4 Complement	25 mg/dL	14 - 44 mg/dL
UA	Normal	Normal
HIV	Nonreactive	Nonreactive
SARS-CoV-2	Negative	Negative

The initial electrocardiography (ECG) was unremarkable; however, the transthoracic echocardiogram (TTE) revealed a trileaflet aortic valve with trivial central aortic insufficiency, along with dilatation of the aortic annulus, sinotubular junction, and ascending aorta with normal biventricular function. Subsequently, computed tomography angiography (CTA) demonstrated segmental thickening involving the aortic root, ascending, descending, and proximal abdominal aorta. Notably, the arterial wall thickening extended into the takeoff and proximal LM coronary artery, causing 50% narrowing. Surprisingly, whole-body positron emission tomography-computed tomography (PET/CT) scan showed no abnormal uptake from the aorta.

Left heart catheterization (LHC) revealed a discrete 70% lesion (fibrotic, based on intravascular ultrasound findings) involving the ostium of the LM. Otherwise, the left anterior descending (LAD) artery, left circumflex artery (LCX), and right coronary artery (RCA) appeared normal (Video 1).

**Video 1:** Initial left heart catheterization The left coronary angiography film was obtained from the right anterior caudal view, and the right coronary angiography film was captured from the left anterior cranial view. Both catheterization films revealed severe left main stenosis, as indicated by yellow arrows.

**Figure 1:**
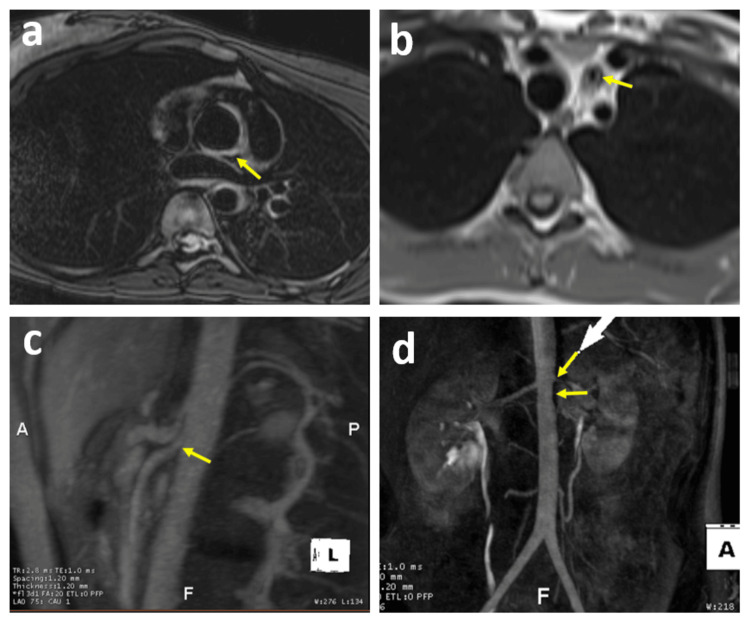
Magnetic resonance angiography (MRA) of the chest and abdomen The MRA findings reveal ascending aortic wall thickening, left common carotid artery wall thickening and narrowing, proximal superior mesenteric artery narrowing, and left renal artery stenosis, as depicted by the yellow arrows in images a, b, c, and d.

Subsequently, the patient underwent magnetic resonance angiography (MRA) of the chest and abdomen, which revealed multifocal areas of vessel wall edema and fibrosis involving the thoracic and abdominal aorta and their branches, indicative of active inflammation. The edema and thickening were noted from the aortic root extending into the proximal ascending aorta, involving the ostium of the RCA without stenosis, and the LM coronary artery causing severe stenosis. Additionally, the imaging revealed diffuse severe thickening and narrowing of the left common carotid artery, moderate narrowing in the left renal artery, and mild to moderate narrowing in the proximal segment of the superior mesenteric artery (Figure 1). The ostium of the left subclavian artery, the mid-descending aorta, and the juxtarenal aorta were also involved in the process.

The multidisciplinary team approach involved a cardiologist, cardiac surgeon, dermatologist, and rheumatologist. Due to the extensive aortitis and involvement of the proximal branches, she was diagnosed with TA and the left hip lesion was presumed to be pyoderma gangrenosum. Initial management involved high-dose methylprednisolone (1000 mg) for three days, followed by a transition to prednisone. Other medications included infliximab, methotrexate (MTX), aspirin, clopidogrel, and metoprolol. Plans for coronary artery bypass grafting (CABG) were deferred due to the active inflammation.

Approximately four months after the initial diagnosis, a follow-up MRA revealed normal biventricular size and function without gadolinium enhancement. The severe narrowing of the left common carotid artery was unchanged. There was some improvement in the thickening of the proximal ascending and infrarenal aorta. The proximal left renal artery narrowing was improved as well.

Subsequently, the prednisone was tapered off and the patient remained on MTX and infliximab.

MTX and infliximab were discontinued around eight months later and the patient was transitioned to tocilizumab. Following this, she underwent successful percutaneous coronary intervention (PCI), and two overlapping drug-eluted stents were placed in the LM artery for the 80% ostial stenosis using the snorkeling technique with approximately 10 mm stent extending into the aorta. The medications at this point involved clopidogrel, aspirin, and tocilizumab. Six months post-PCI, clopidogrel was discontinued, and the patient continued with aspirin and tocilizumab.

About one month after the cessation of clopidogrel, the patient experienced chest pain and cardiac arrest attributed to ventricular fibrillation. Immediate cardiopulmonary resuscitation was initiated, followed by prolonged support using the LUCAS device for two hours, and subsequently by the implementation of venoarterial extracorporeal membrane oxygenation (VA-ECMO). During the LHC repeated attempts to engage the LM proximal stent opening failed due to complete thrombosis of the stent. Selective angiography and coronary filling through the side struts of a protruding stent revealed about 20% in-stent restenosis of the LM (Video 2). The patient subsequently underwent successful balloon angioplasty by dilating the side strut of the stent, resulting in thrombolysis in myocardial infarction (TIMI) 3 reflow.

**Video 2:** Postcardiac arrest left heart catheterization The left capture, an aortic root angiography, reveals proximal snorkeling stent thrombosis and late contrast filling through the side strut. The middle film, captured from the right anterior cranial view, displays catheter engagement into the side struts. The right catheterization film, obtained from the right anterior caudal view demonstrates reflow (TIMI 3) following balloon angioplasty of the side struts.

The patient remained in persistent cardiogenic shock for approximately three weeks, requiring VA-ECMO, followed by an Impella, with intermittent use of pressors for an ejection fraction (EF) of 20-25%. Multiple attempts to wean from the Impella were unsuccessful. A PET myocardial perfusion and viability study indicated hibernation of the entire anterior wall, septum, and lateral wall, supporting the need for revascularization. Subsequently, the patient underwent CABG with left internal mammary artery to LAD and saphenous venous graft to the obtuse marginal artery. Despite these interventions, cardiogenic shock persisted. Eventually, the Impella device was explanted, and a durable left ventricular assist device (LVAD) was implanted as a bridge to recovery or heart transplantation.

## Discussion

The patient's presentation and imaging findings were consistent with TA. Managing LM ostial lesions in this condition is challenging due to a lack of highly validated recommendations. While CABG is generally favored for better outcomes in terms of restenosis, PCI following appropriate immunotherapy shows comparable results to CABG in TA, though these recommendations are not specific for the LM disease. Our patient received appropriate immunotherapy, and repeat MRA showed some improvement of the arterial lesions. Cardiac arrest occurred one month after discontinuation of clopidogrel. Post-arrest LHC revealed complete thrombosis at the proximal stent opening, with 20% intracoronary stenosis, suggesting stent thrombosis as the likely cause of cardiac arrest rather than restenosis. The unique aspect of this case is that the late thrombosis occurred in the intraaortic portion of the stent. Several risk factors could precipitate stent thrombosis in this location including the stent material itself in the setting of absent prothrombotic subendothelial constituents, possible TA exacerbation, and definitely discontinuation of the clopidogrel. This case underscores the potential importance of CABG for treating LM disease in TA, even after adequate immunotherapy. The optimal duration of dual antiplatelet therapy (DAPT) in this condition remains unclear but should likely exceed six months.

The snorkeling technique is recognized for preventing coronary obstruction during the transcatheter aortic valve replacement (TAVR) procedure. However, there is limited documentation on snorkeling stent thrombosis associated with this procedure, with only one reported case resulting in a fatal outcome post-TAVR [5].

History and epidemiology

Initially thought to affect only women in East Asia, TA is now recognized globally, affecting both genders, with variations in clinical presentation across different regions. The predominance of the disease in females compared to males tends to decrease from East Asia to the West [6]. Published descriptions of this arteritis date back to 1830 [7]. Yamamoto reported a case of a 45-year-old man with persistent fever who developed impalpable upper limb and carotid pulses, accompanied by weight loss and dyspnea [8]. In 1905, Takayasu, a professor of ophthalmology in Japan, presented a case of a 21-year-old woman with characteristic fundal arteriovenous anastomoses [9]. These early reports created the foundation for our understanding of this complex condition.

TA is a rare disease with limited data on its incidence, likely due to its rarity. It is believed to be more prevalent among Asian populations, given the high number of reports from these regions. Available data suggest varying incidence across different regions, with estimates ranging from 0.4 to 3.4 per million [10]. Studies on the prevalence of TA are limited; most are single-center studies with small patient numbers. Nationwide studies in Japan and Kuwait estimated prevalence rates of 40 per million and 7.8 per million, respectively. European studies reported lower prevalence rates, ranging from 0.9 to 25.2 per million. The true prevalence of TA may be higher due to underdiagnosis and misdiagnosis, highlighting the need for increased awareness and accurate diagnostic criteria [10].

TA primarily affects the aorta and its large branches, with pulmonary artery involvement in about 70% of cases [11]. Coronary artery involvement is seen in 9-10% of cases, though one coronary computed tomography angiography-based study reported a 53% prevalence of coronary artery lesions [12-14]. Coronary artery involvement was first characterized in 1951 in a postmortem study describing coronary stenosis and occlusions [15]. Matsubara et al. provided a thorough report of coronary artery lesions in TA describing three patterns: type 1, stenosis or occlusion of coronary ostia or proximal segments; type 2, diffuse or focal coronary lesions that may involve all the epicardial branches or only focal segments; type 3, coronary artery aneurysms [16].

Pathophysiology

The etiology and pathophysiology of TA remain poorly understood [17,18]. Both humoral and cell-mediated mechanisms contribute to its pathogenesis, leading to inflammation and tissue damage [19]. Inflammation and intimal proliferation are key mechanisms in the development of stenosis, occlusion, and destruction of the elastic and muscular layers, resulting in aneurysms and dissections [17]. Molecular mimicry between host and microbial heat-shock proteins may drive innate immune responses. Additionally, pro-inflammatory cytokines such as IL-6 and C-C chemokine ligand 5 (RANTES) induce matrix metalloproteinase production, which destroys elastic fibers in the arterial wall. Targeting cytokine production may offer a promising therapeutic strategy in TA [19].

Clinical presentation

The clinical presentations of TA are diverse and do not always follow the traditional "triphasic" pattern of constitutional symptoms, followed by vascular symptoms progressing into significant ischemic events. A study of 275 TA patients revealed a wide range of symptoms, including constitutional symptoms, carotidynia, vascular-associated symptoms, major ischemic events, and asymptomatic presentations [20,21].

TA's clinical manifestations range from subtle signs like absent pulses or bruits to severe neurological impairments. Early stages often involve non-specific symptoms such as fever, nocturnal sweating, fatigue, weight loss, joint and muscle pain, and mild anemia [22]. As inflammation progresses, stenoses develop, leading to distinct features like collateral circulation formation. Stenosis is the most common lesion, often bilateral, and as the disease advances, symptoms and complications become severe, resulting in significant morbidity. Common sequelae include retinopathy, secondary hypertension, aortic aneurysm, and regurgitation [23]. Coronary artery involvement can present as chest pain in the context of acute coronary syndrome or sudden cardiac death in some cases. Acute coronary events may progress into heart failure in late stages as a result of ischemic cardiomyopathy [1].

Diagnosis

The diagnosis of TA is based on clinical findings supported by imaging abnormalities, as there are no definitive laboratory tests to confirm the disease. Acute phase reactants such as C-reactive protein (CRP) and erythrocyte sedimentation rate (ESR) are neither specific nor sensitive and do not correlate well with disease activity. Imaging modalities like MRA or CTA are crucial for diagnosing and evaluating the extent of the disease [24]. MRA is often preferred due to the lack of radiation exposure and low risk of contrast-induced nephropathy. Color Doppler ultrasound can monitor disease activity in internal carotid and subclavian arteries, while coronary angiography is essential for diagnosing coronary artery involvement. PET has gained popularity for detecting vascular wall uptake but remains investigational [25]. Biopsy and histopathology can support the diagnosis but are not mandatory [26].

The 2022 ACR/European Alliance of Associations for Rheumatology (EULAR) classification considers age ≤60 at diagnosis and imaging findings consistent with vasculitis absolute requirement for the diagnosis of TA [26]. Additional criteria include female gender, angina pectoris, claudication, vascular bruit, reduced upper extremity pulse, carotid artery abnormalities, systolic blood pressure difference in arms ≥20 mmHg, and specific imaging criteria such as the number of affected arterial territories and involvement of symmetric paired arteries or abdominal aorta with mesenteric and renal arteries. A score of ≥5 is needed for a TA diagnosis [26].

Treatment

The standard treatment for TA involves high-dose glucocorticoids (GCs) to achieve remission. However, relapses are common, and prolonged GC therapy can cause severe side effects. To mitigate these risks, GC-sparing agents are often used. MTX is a commonly used immunosuppressive medication that, when combined with GCs, can induce remission in significant proportion of TA patients. Biologic agents like tocilizumab, targeting the IL-6 receptor, have shown promising results in managing refractory TA, reducing disease activity and GC doses. Other biologics, such as tumor necrosis factor inhibitors (TNFi), have been used with mixed results in resistant cases [27].

Treating coronary artery involvement is challenging due to lesion locations, particularly at the ostia of arteries. Interventions should be avoided during active inflammation unless it is an emergency [28,29]. The typical revascularization method is CABG [29]. While some reports indicate successful PCI in these patients, data show an increased risk of restenosis after PCI [30,31]. An interesting review article has demonstrated the superiority of CABG over PCI regarding major adverse cardiovascular events (MACE), especially during the acute phase of TA. The same source reported that when intervention is performed during the remission of TA, PCI and CABG-related MACE outcomes are similar [28]. Another study replicated better outcomes for CABG than PCI, suggesting chronic inflammation predisposes to high restenosis rates after PCI [4]. Coronary stenting should be considered for patients who refuse CABG, are poor surgical candidates, or in emergencies [32,33]. Reports on revascularization methods for specifically LM coronary artery stenosis are conflicting [34-36].

## Conclusions

To the best of our knowledge, this is the first report of snorkeling stent thrombosis in the context of TA. In young female patients, TA should be considered when presentation involves anginal symptoms, especially in the setting of coronary artery ostial involvement, as it can be life-threatening. Managing ostial stenotic lesions is challenging. Currently, there is no well-validated consensus on revascularization modalities for these patients. Given TA's fluctuating course and difficulty in assessing disease activity, PCI may be associated with increased risks of thrombosis and restenosis during exacerbations. This case potentially indicates the importance of preferring CABG for LM revascularization in TA and suggests a possible need for longer duration of DAPT.
